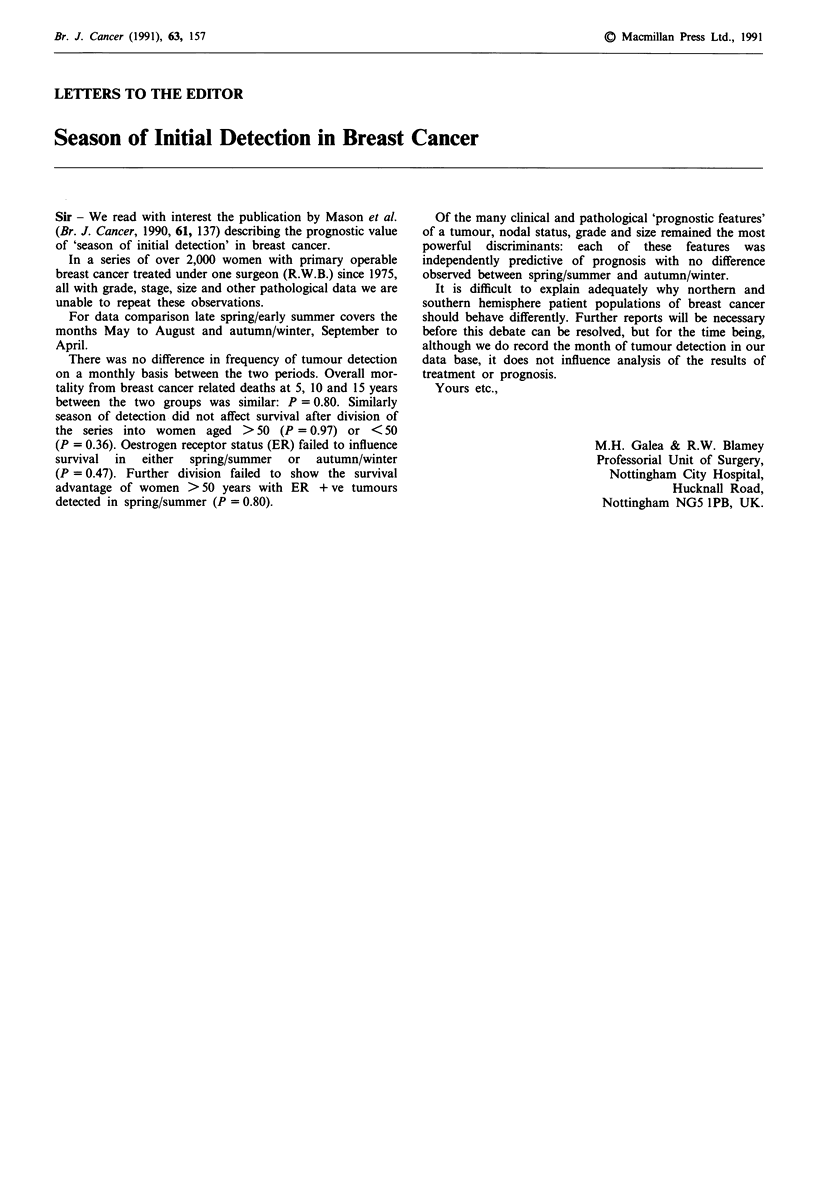# Season of initial detection in breast cancer.

**DOI:** 10.1038/bjc.1991.33

**Published:** 1991-01

**Authors:** M. H. Galea, R. W. Blamey


 © Macmillan Press Ltd., 1991

### LETTERS TO THE EDITOR

### Season of Initial Detection in Breast Cancer

**Sir** – We read with interest the publication by Mason *et al.* (*Br. J. Cancer*, 1990, **61,** 137) describing the prognostic value of 'season of initial detection' in breast cancer.

In a series of over 2,000 women with primary operable breast cancer treated under one surgeon (R.W.B.) since 1975, all with grade, stage, size and other pathological data we are unable to repeat these observations.

For data comparison late spring/early summer covers the months May to August and autumn/winter, September to April.

There was no difference in frequency of tumour detection on a monthly basis between the two periods. Overall mortality from breast cancer related deaths at 5, 10 and 15 years between the two groups was similar: $P = 0.80$. Similarly season of detection did not affect survival after division of the series into women aged $>50$ ($P = 0.97$) or $<50$ ($P = 0.36$). Oestrogen receptor status (ER) failed to influence survival in either spring/summer or autumn/winter ($P = 0.47$). Further division failed to show the survival advantage of women $>50$ years with ER +ve tumours detected in spring/summer ($P = 0.80$).

Of the many clinical and pathological 'prognostic features' of a tumour, nodal status, grade and size remained the most powerful discriminants: each of these features was independently predictive of prognosis with no difference observed between spring/summer and autumn/winter.

It is difficult to explain adequately why northern and southern hemisphere patient populations of breast cancer should behave differently. Further reports will be necessary before this debate can be resolved, but for the time being, although we do record the month of tumour detection in our data base, it does not influence analysis of the results of treatment or prognosis.

Yours etc.,

M.H. Galea & R.W. Blamey
Professorial Unit of Surgery,
Nottingham City Hospital,
Hucknall Road,
Nottingham NG5 1PB, UK.